# Body dysmorphic disorder and depression among male undergraduate students in a Malaysian University

**DOI:** 10.3389/fpsyt.2022.977238

**Published:** 2022-09-27

**Authors:** Waye Hann Kang, Min Yee Loo, Xue Min Leong, Yi Fan Ooi, Wen Qi Teo, Teng Jun Neoh, Wei Chih Ling

**Affiliations:** ^1^Department of Medicine, Faculty of Medicine and Health Sciences, University Tunku Abdul Rahman, Kajang, Malaysia; ^2^Department of Preclinical Sciences, Faculty of Medicine and Health Sciences, University Tunku Abdul Rahman, Kajang, Malaysia

**Keywords:** body dysmorphia, depression, Malaysian students, height dissatisfaction, obsessive-compulsive disorder

## Abstract

**Introduction:**

Body dysmorphic disorder (BDD) and depression have been reported to be both prevalent among young people worldwide, resulting in serious implications in their quality of life and social functioning. This is worrying especially in men where mental disorders are often overlooked and under-researched. This study aims to determine the proportion of male university students with symptoms suggestive of body dysmorphic disorder and depression, as well as their perception on their body image.

**Methods:**

In this cross-sectional study, 1,308 male students between the aged of 17–26 years in a private university in Malaysia *via* self-administered online questionnaire comprising the Patient Health Questionnaire (PHQ)-9, the Body Dysmorphic Disorder Questionnaire (BQQD) and the Body Self-Image Questionnaire (BSIQ), in addition to their sociodemographic parameters. Data analyses were performed with Mann Whitney test, chi square test and Fisher's exact test.

**Results:**

3.3 and 54.2% of the students had symptoms suggestive of BDD and depression respectively, with up to 9.02% of the students reporting having moderate to severe depression. There was a significant association between BDD symptoms and students staying alone, whilst depression was significantly associated with studying in the rural campus. Furthermore, a significant association was demonstrated between presence of BDD and depression symptoms. Most of the students were dissatisfied with their whole body, especially their height.

**Conclusion:**

The proportion of BDD and depression symptoms among male students in our university is quite high. Universities and the public health sector should develop better support service targeting male university students.

## Introduction

Body dysmorphic disorder (BDD), one of an obsessive-compulsive spectrum disorder, is often described as having distressing or impairing obsessions with perceived physical imperfections that are usually unobservable or appear only slight to others (1–3). Individuals with BDD often have a negative perception on their own body image that leads to significant negative emotions thus affecting their daily functioning (4). BDD in adults usually leads to high rates of social and occupational dysfunction whereas BDD in youth is associated with a poorer academic performance, social withdrawal and higher school dropping out rates (2). Despite the increasing prevalence of BDD among young people worldwide (4–11) and the serious implications toward their quality of life and social functioning, there is still a lack of data on BDD in the general population of Malaysia, especially among young male adults.

Depression is characterized by persistent sadness, anhedonia, trouble sleeping, changes in appetite, loss of energy and difficulty concentrating and is common among university students (12). In Malaysia, it was reported up to 70% of university students admitting that they have symptoms suggesting of depression (13–16). Aside from affecting the academic achievement of students (17), untreated depression may also lead to increased psychological morbidity and other mental disorders (18).

Women are known to be more prone to suffer from internalizing mental disorder, such as major depression and eating disorders (19), while mental illnesses in men are often overlooked and untreated, resulting in higher morbidity and mortality rates (20). Although suicide has been the leading cause for death among young men in several countries, the mental health of this group is generally overlooked (21). This implies that there is an urgent need to study the epidemiology of mental disorders among male adolescence. The current study aims to determine the proportion of symptoms suggesting of BDD and depression among male undergraduate students in a local university as well as their associated factors. Furthermore, this study examines the body self-image perception among these students. We hypothesize that a high number of male Malaysian university students may be a risk of depression and BDD.

## Methods

### Study design

This was a single-center cross-sectional study conducted between 11 November 2021 till 20 January 2022. The target population of this study was male foundation students and undergraduates aged from 17 to 26 years from both Universiti Tunku Abdul Rahman (UTAR) Sungai Long Campus and Kampar campus. Ethics approval was obtained before the initiation of the study.

The sample size was calculated based on the formula for estimation for a proportion, $n=\frac{Z^{2}P\;\left(1-P\right)}{d^{2}}$ where n is the sample size, Z is standard normal deviate 1.960, d is the precision of 0.03, and p is the pre-study estimate of depression among Malaysian medical students as reported by Shamsuddin et al. (13).

A random sampling method was used where 2,149 participants were randomly selected from the list of 10,747 male undergraduate students registered in the university, which represented 20% of the total population. All male foundation students and undergraduates from Universiti Tunku Abdul Rahman, between the ages of 17–26 years and students were included in the study and excluded if they refused to participate in this study or were not able to provide informed consent. Students agreeable to participate in the study were given a self-administering online questionnaire. A total of 1,308 students agreed to enroll in the study, showing a calculated response rate of 60.8%, whilst 24 students decline to participate and the remaining 817 students did not respond within the stipulated timeframe.

### Measures

The questionnaire consists of 7 sections: (i) sociodemographic parameters, (ii) Patient Health Questionnaire-9 (PHQ-9), (iii) Body Dysmorphic Disorder Questionnaire (BDDQ), and (iv) Body Self-Image Questionnaire-Short Form (BISQ-SF). The 6 socio-demographic parameters included age, gender, faculty, household members, history of mental illness, history of recreational drug abuse.

The Patient Health Questionnaire-9 (PHQ-9) is widely used to access the respondents' depression severity levels based on a four-point Likert scale rated from 0 (Not at all) to 3 (nearly every day). This section consisted of 9 questions and the total sum up scores was classified according to the following categories in terms of depression severity level: none or minimal, mild, moderate, moderately severe and severe. This depression subscale has been published in previous studies, showing a Cronbach alpha of 0.86–0.89 respectively (22).

The BDDQ is a self-report screening tool for BDD and consists of 4 questions asking about the concern of the physical appearance and a total score of 4 indicates a possibility of BDD. Phillips et al. reported a sensitivity and specificity of this scale of 100 and 93% respectively (23).

The Body Self-Image Questionnaire-Short Form (BSIQ-SF) is used to access the participant's perceptions toward their body image. This questionnaire has 21 Likert scale questions which each classified into 4 different domains which included negative affect, attractiveness evaluation, physical functionality awareness and height dissatisfaction. Higher dissatisfaction toward body and height is usually indicated by higher scores in negative affect and height dissatisfaction. On the contrary, a higher score in attractiveness evaluation and physical functionality awareness points toward the individual having a higher body satisfaction and more likely to maintain good physical functionality. The results were accessed using 5-point Likert scale included: “Not at all true of myself,” “slightly true,” “about halfway true,” “mostly true,” and “completely true.” The BSIQ-SF has been validated for local use in Malaysia (24).

### Statistical analysis

The data were analyzed with SPSS version 22.0 (IBM, Chicago, USA). Descriptive statistics are presented as frequency and percentage for categorical data and as mean and standard deviation for continuous variable if they are normal distributed or as median and interquartile range (IQR) if they are not normal distributed. In addition to it, all quantitative data was assessed for normal distributions in this study. Chi-square or Fisher's exact test were used to analyse categorical variables. On the other hand, Mann-Whitney test or Kruskal-Wallis test was employed to look for the relationship between categorical data (sociodemographic parameters) and continuous data (BDDQ/ PHQ-9/ BSIQ-SF). For continuous data, Spearman's correlation test was used to analyse the correlation between age and BSIQ scores. For logistic regression, a backward stepwise approach is chosen to determine statistically significant independent variables.

The statistical significance level for all inferential test was set at *P* < 0.05.

## Results

### BDD among UTAR male undergraduate students and its associated factors

Based on the BDDQ questionnaire, approximately 3.3% out of the 1,308 male undergraduate students reported symptoms suggestive of BDD. Among all the socio-demographic parameters, there is a significant association between BDD symptoms and students staying alone (x^2^ = 5.726, *p* = −0.026) (Table 1). There is no significant association between BDD symptoms and students studying medicine and health sciences (x^2^ = 0.012, *p* = 0.914) with only 7.5% out of 43 the students with BDD symptoms were from the faculty of medicine and health sciences (Table 1).

**Table 1:** Socio-demographic parameters of UTAR male undergraduate students according to BDD and depression status.

**Variable**	**BDD**	**No BDD**	***p*** **value**	**Depression**	**No depression**	***p*** **value**
	**(*n* = 43)**	**(*n* = 1,265)**		**(*n* = 709)**	**(*n* = 599)**	
**Campus**			0.28*^a^*			0.002^a^
Sungai Long Campus	18	640		330	328	
Kampar Campus	25	625		379	271	
**Faculty**			0.12*^b^*			0.129*^a^*
Faculty of Accountancy and Management	1	97		51	47	
Faculty of Arts and Social Science	6	68		49	25	
Faculty of Business and Finance	3	164		92	75	
Faculty of Creative Industries	4	40		27	17	
Faculty of Engineering and Green Technology	3	43		28	18	
Faculty of Information and Communication	9	149		89	69	
Faculty of Medicine and Health sciences	4	83		41	46	
Faculty of Science	6	86		51	41	
Institute of Chinese Studies	0	4		2	2	
Faculty of Engineering and Science	4	314		152	166	
Center for Foundation Studies	3	217		127	93	
**Staying alone**			0.026^a^			0.125*^a^*
Yes	8	104		67	45	
No	35	1,161		642	554	
**Family history of mental illness**			0.126*^b^*			0.051*^a^*
Yes	3	35		26	12	
No	40	1230		683	587	
**History of recreational drug use**			0.791*^b^*			0.408*^b^*
Yes	0	7		3	4	
No	43	1,258		706	595	
**Family history of recreational drug use**			0.265*^b^*			0.151*^b^*
Yes	1	13		10	4	
No	42	591,252		699	595	

a Chi square;

b Fisher's Exact test.

Only 793 (60.6%) of all the respondents are worried about their appearance, and among these respondents, 60.4% of them wished they could think about their appearance less. 39.6% of these respondents were dissatisfied with their whole body, while 37.1% were unhappy about their face. In addition, 46.4% of the respondents were concerned that they did not look thin enough or might look too fat, and 30.6% felt that this often upset them a lot.

Due to these dissatisfactions, 127 (16.0%) out of the 793 students admitted that this had gotten in the way of their social activities and 50 (6.3%) claimed that it has caused problems with their school, work, or other activities. Only 2.9% of these 793 students spent more than 3 h thinking about their look, followed by 7.9% spending 1–3 h per day and 89.2% spent less than an hour.

With multivariate logistic regression, after adjusting for family history of mental illness or recreational drug use, personal history of recreational drug use, as well as location of campus, the only significant predictor for BDD symptoms is staying alone (OR = 2.551, 95%CI 1.153–5.649, *p* = 0.021).

### Depression symptoms among UTAR male undergraduate students and its associated factors

Approximately 54.2% of students reported to have symptoms suggestive of depression in the PHQ-9 questionnaire where 9.02% had symptoms suggestive moderately severe to severe depression while 13.23 and 31.96% had moderate and mild depressive symptoms respectively. No significant association between most of the socio-demographic parameters was seen except for location of the campus (x^2^ = 8.762, *p* = −0.003). Almost 50% of male students studying medicine or health sciences had symptoms suggestive of depression, but this only constitutes 6% of all the students claiming to have depression symptoms. There was also no significant association between medical and health sciences students with symptoms of depression (x^2^ = 2.195, *p* = 0.138) (Table 1).

The median and interquartile range for the PHQ-9 score was 5 (7); the mean and IQR PHQ-9 score for students with symptoms suggestive of depression is significantly higher than those without symptoms (U = 0, *p* < 0.001). Among all the variables, students from the Kampar campus and with a family history of recreational drug use had significantly higher PHQ-9 median scores respectively compared to students from Sungai Long campus or without family history of recreational drug use (U = 199,188, *p* = 0.031; U = 5,546.5, *p* = 0.012). The most frequent symptoms that the respondents had were issues with energy levels (71.5%), followed by little interest or pleasure in doing things (68.4%) and feelings of down and depressed and hopelessness (55.2%) (Table 2).

**Table 2:** Symptoms suggestive of depression and their frequency among UTAR male undergraduate students in the PHQ-9 questionnaire.

	**Not at all**	**Several Days**	**More than half the days**	**Nearly every day**
	**(%)**	**(%)**	**(%)**	**(%)**
Little interest or pleasure in doing things	413 (31.6%)	635 (48.5)	173 (13.2)	87 (6.7%)
Feeling down, depressed, or hopeless	586 (44.8%)	537 (41.1%)	124 (9.5%)	61 (4.7%)
Trouble falling or staying asleep, or sleeping too much	594 (45.5)	433 (33.1)	159 (12.2)	122 (9.3)
Feeling tired or having little energy	373 (28.5)	585 (44.7)	227 (17.4)	123 (9.4)
Poor appetite or overeating	850 (65)	303 (23.2)	96 (7.3)	59 (4.5)
Feeling bad about yourself – or that you are a failure or have let yourself or your family down	654 (50)	396 (30.3)	163 (12.5)	95 (7.3)
Trouble concentrating on things	680 (52)	379 (29)	167 (12.8)	82 (6.3)
Moving or speaking so slowly that other people could have noticed or so fidgety or restless that you have been moving a lot more than usual	980 (74.9)	239 (18.3)	63 (4.8)	26 (2)
Thoughts that you would be better off dead, or thoughts of hurting yourself in some way	1,003 (76.7)	196 (15)	71 (5.4)	38 (2.9)

With multivariate logistic regression, after adjusting for family history of mental illness or recreational drug use, personal history of recreational drug use, as well as status of living alone, the only significant predictor for symptoms suggestive of depression is location of campus (OR = 1.397, 95%CI 1.122–1.736, *p* = 0.003).

### Perception on body self-image and its correlation between depression and BDD among UTAR male undergraduate students

25.7% of all the 1,308 students did not think they look good in their clothes. Most of the students were unhappy with their height, with 23.8% students often expressed their wish to be taller, 17.9% wished that they could be taller and 13.1% expressed that they would like their body better if they were taller. On the contrary only 6.7% of the students thought that they were overweight and 9.2% wished they were thinner (Table 3).

**Table 3:** Body self-image perception of UTAR male undergraduate students according to the BSIQ.

	**Not at all**	**Slightly**	**About halfway**	**Mostly**	**Completely**
	**(%)**	**(%)**	**(%)**	**(%)**	**(%)**
**Negative affect**					
I think my body is unattractive	260 (19.9)	472 (36.1)	334 (25.5)	185 (14.1)	57 (4.4)
I think my body looks fat in clothes	612 (46.8)	260 (19.9)	213 (16.3)	161 (12.3)	62 (4.7)
My naked body makes me feel sad	594 (45.4)	323 (24.7)	207 (15.8)	121 (9.3)	63 (4.8)
Being around good-looking people makes me feel bad about my body	473 (36.2)	301 (23.0)	281 (21.5)	171 (13.1)	82 (6.3)
My body is overweight	754 (57.6)	191 (14.6)	158 (12.1)	118 (9.0)	87 (6.7)
I feel depressed about my body	700 (53.5)	289 (22.1)	203 (15.5)	81 (6.2)	35 (2.7)
I wish I were thinner	618 (47.2)	221 (16.9)	193 (14.8)	156 (11.9)	120 (9.2)
Most days I feel bad about my body	703 (53.7)	301 (23)	195 (14.9)	63 (4.8)	46 (3.5)
**Attractive awareness**					
I look good in clothes	336 (25.7)	294 (22.5)	338 (25.8)	216 (16.5)	124 (9.5)
My body is healthy	79 (6.0)	204 (15.6)	415 (31.7)	426(32.6)	184 (14.1)
I'm usually well dressed	236 (18.0)	352 (26.9)	426 (32.6)	221(16.9)	73 (5.6)
My body looks good	228 (17.4)	393 (30.0)	465 (35.6)	165 (12.6)	57 (4.4)
My body is in shape	267 (20.4)	408 (31.2)	420 (32.1)	162 (12.4)	51 (3.9)
Having a well-proportioned body is important to me	111 (8.5)	233 (17.8)	309 (23.6)	430 (32.9)	225 (17.2)
**Physical functionality awareness**					
I pay careful attention to my face and hair, so that I will look good	199 (15.2)	326 (24.9)	344 (26.3)	306 (23.4)	133 (10.2)
I feel better about my body when I'm fitter	122 (9.3)	167 (12.8)	272 (20.8)	442 (33.8)	305 (23.3)
Body size matters to me	277 (21.2)	406 (31.0)	436 (33.3)	153 (11.7)	36 (2.8)
The way I feel about my body improves when I exercise regularly	90 (6.9)	220 (16.8)	313 (23.9)	444 (33.9)	241 (18.4)
**Height dissatisfaction**					
I often wanted to be taller	237 (18.1)	225 (17.2)	224 (17.1)	311 (23.8)	311 (23.8)
I wish I were a different height	366 (28)	248 (19)	214 (16.4)	246 (18.8)	234 (17.9)
If i were a different height, I'd like my body better	369 (28.2)	236 (18.1)	273 (20.9)	257 (19.6)	172 (13.1)

Respondents with symptoms suggestive of depression based on the PHQ-9 scores, had a significantly higher scores in the negative affect (U = 130,792, *p* < 0.001) and height dissatisfaction (U = 162,738, *p* < 0.001) domains, while their scores in the attractive awareness domain was significantly lower (U = 194,876, *p* = 0.01). Similarly, respondents with symptoms of BDD had significantly higher scores in the negative affect (U = 7,354, *p* < 0.001) and height dissatisfaction domains (U = 18,526, *p* < 0.001) but a significantly lower score in the physical functionality domain (U = 20,460, *p* = 0.005).

There was a significant positive correlation between the PHQ-9 score and the negative affect score (r (1306) = 0.373 *p* < 0.001), height dissatisfaction score (r (1306) = 0.193 *p* < 0.001), and the physical functionality score (r (1306) = 0.086 *p* = 0.002) respectively. However, there was a significant negative correlation between PHQ-9 score and attractive awareness score (r (1306) = −0.089, *p* = 0.001).

## Discussion

### BDD among UTAR male undergraduate students and its associated factors

The proportions of students at risk of having BDD was almost similar to previous studies which reported 0.6–12.3% of their male cohort having BDD (4–11). Once again, the application of different assessment tools to determine BDD in different studies could have resulted in the large range of prevalence observed. Most of the respondents in our study were dissatisfied with their whole body and face whereas respondent from other studies were more concern with their hair, face, weight and skin (4, 7–9, 25). Separately, there is a significant association between staying alone and BDD, as individuals with BDD often have poor psychosocial functioning and avoids social interaction (26).

Most of our respondents were dissatisfied with their height, as evident by the BSIQ scores. This was in line with other previous literature that reported Asian boys and men are more discontent with their height (27). Men often perceive height as an important component of masculinity. Taller men are thought to be more attractive, more intelligent and more confident (28, 29). A high dissatisfaction in height among our cohort may be due to the fact that a person's height cannot be altered easily without invasive or potentially dangerous intervention (30). However, we did not assess whether height dissatisfaction results in disability or impairment among our respondents and we did not include screening for eating disorders in our study, as this is closely related to BDD. In this, we also believe that height dissatisfaction should be spelled out clearly as a “body area” in the BDDQ owing to its high proportion among young Asian men and possibly improving the sensitivity of the BDDQ questionnaire.

### Depressive symptoms among UTAR male undergraduate students and its associated factors

Our study reported up to 54.2% who may be at risk of depression due to the presence of symptoms. This is comparable to other local studies which had reported 33.4–74.4% in their male cohort (13–16). The application of various screening tools for depression in previous studies may explain the large range of prevalence observed whereby most of our male students reported mild depression symptoms, compared to the previous studies where most of their male students had moderate depressive symptoms. However, the global pooled prevalence of depression among both male and female college students were reported to be 33.6% (31).

Interestingly, there were significantly more students at risk of depression in the Kampar campus, which is located in a rural area, contrary to prior studies (15). Our study is the first to report this observation as previous literatures have pointed out a higher prevalence of mental health problems in adolescents residing in urban areas, suggesting a detrimental effect of urbanicity among adolescents (32–34).

Prior studies have reported a higher degree of depression among medical students (31). In our cohort, up to 46.5% of the male undergraduate students enrolled in medical and health sciences had depressive symptoms. The results from our finding was almost similar to previous local studies which reported that 33.3–74.4% Malaysian medical and health science students exhibited symptoms of depression (15, 35–38), suggesting that medical students were more vulnerable to develop depressive symptoms compared to other students (39). This may largely due to the fact that curriculum for medical and health science students are more intense and complex compared to the other students (35, 37, 40, 41). However, no significant association was seen between enrolling in medical and health sciences and depression.

### BDD and depression

BDD and depression symptoms among our participants were significantly associated with each other (x^2^ = 9.099, *p* = 0.003). There is conflicting literature regarding the association of BDD and depression where some literature reported individuals with BDD has a 2.3–4.2 times higher risk for co-existing neuropsychiatric disorders especially depression (11, 42), whilst Pimenta et al. commented that there were no association between BDD and depression in their study (43). However, both studies were conducted in both men and women, while our study only focuses on young men. This association can be partially explained by the higher susceptibility of men with BDD exhibiting negative thought and behavior patterns which increases the vulnerability of developing depression (42).

### Strengths and limitations

To our knowledge, this study is the first in the country to determine the proportion of students with symptoms suggestive of body dysmorphic among young adults as well as their perception on their self-image. However, our sample population consists mainly of students of Chinese ethnicity. Hence, our findings may not be generalized to the other universities in the country. Furthermore, both the PHQ-9 and BDIQ only serve as screening tools for depression and BDD and the diagnosis of both mental disorders require a more structured interview by a trained healthcare provider. A mediator or moderator modeling would be more superior to determine the association between the independent variables and the presence of BDD and depression symptoms. In addition to this, the proportion of depressive symptoms could be much higher than expected as the study was conducted during the COVID-19 pandemic where the movement restriction order was being implemented.

## Conclusion

There is a high proportion of young male university students who is at risk of developing depression and BDD. Majority of our male university students are dissatisfied with their height, where the level of dissatisfaction significantly correlates with the severity of depressive symptoms. This implicates the dire need for university authorities to take a proactive approach to screen for depression and BDD among university students, as well as to educate them on mental health resilience.

## Data availability statement

The original contributions presented in the study are included in the article/supplementary material, further inquiries can be directed to the corresponding author.

## Ethics statement

The studies involving human participants were reviewed and approved by Science and Research Ethics Committee, University Tunku Abdul Rahman. The patients/participants provided their written informed consent to participate in this study.

## Author contributions

WK and WL have made substantial contributions to conception and design of the study, interpretation of the data, and as well as critical revision of the manuscript. ML, XL, YO, TN, and WT have been involved in acquisition of data, analysis and interpretation of data as well as early drafting of the manuscript. All authors have given final approval of the version to be published.

## Conflict of interest

The authors declare that the research was conducted in the absence of any commercial or financial relationships that could be construed as a potential conflict of interest.

## Publisher's note

All claims expressed in this article are solely those of the authors and do not necessarily represent those of their affiliated organizations, or those of the publisher, the editors and the reviewers. Any product that may be evaluated in this article, or claim that may be made by its manufacturer, is not guaranteed or endorsed by the publisher.
